# Oligopeptide Transporters of Nonencapsulated Streptococcus pneumoniae Regulate CbpAC and PspA Expression and Reduce Complement-Mediated Clearance

**DOI:** 10.1128/mbio.03325-22

**Published:** 2023-01-10

**Authors:** Courtney D. Thompson, Jessica L. Bradshaw, Wesley S. Miller, Ana G. Jop Vidal, Jorge E. Vidal, Jason W. Rosch, Larry S. McDaniel, Lance E. Keller

**Affiliations:** a Center for Immunology and Microbial Research, Department of Cell and Molecular Biology, The University of Mississippi Medical Center, Jackson, Mississippi, USA; b Mississippi INBRE Research Scholar Program, Mississippi College, Clinton, Mississippi, USA; c Department of Infectious Diseases, St. Jude Children’s Research Hospital, Memphis, Tennessee, USA; UCLA School of Medicine

**Keywords:** nonencapsulated *S. pneumoniae*, *Streptococcus pneumoniae*, innate immunity, oligopeptide transporter, pneumococcus

## Abstract

Streptococcus pneumoniae colonizes the human nasopharynx and causes several diseases. Pneumococcal vaccines target the polysaccharide capsule and prevent most serious disease, but there has been an increase in the prevalence of nonencapsulated S. pneumoniae (NESp). Previously, it was thought that a capsule was necessary to cause invasive disease. NESp strains expressing the oligopeptide transporters AliC and AliD have been isolated from patients with invasive disease. The AliC and AliD oligopeptide transporters regulate the expression of several genes, including choline binding protein AC (CbpAC) (a homolog of PspA), which aids in reducing C3b deposition. It is hypothesized that by altering CbpAC expression, AliC and AliD provide protection from classical complement-mediated clearance by reducing C-reactive protein (CRP) binding. Our study demonstrates that AliC and AliD regulate CbpAC expression in NESp and that AliD found in certain serotypes of encapsulated strains regulates PspA expression. C3b deposition was increased in the NESp Δ*aliD* and encapsulated mutants in comparison to the wild type. NESp strains expressing AliC and AliD have a significant decrease in C1q and CRP deposition in comparison to the Δ*aliC* Δ*aliD* mutant. The complement protein C1q is required for NESp clearance in a murine model and increases opsonophagocytosis. By regulating CbpAC expression, NESp inhibits CRP binding to the bacterial surface and blocks classical complement activation, leading to greater systemic survival and virulence. Due to the increase in the prevalence of NESp, it is important to gain a better understanding of NESp virulence mechanisms that aid in establishing disease and persistence within a host by avoiding clearance by the immune system.

## INTRODUCTION

Streptococcus pneumoniae (pneumococcus) is a Gram-positive bacterium that colonizes the human nasopharynx, residing asymptomatically in healthy individuals (1). After colonization, the pneumococcus can disseminate into other tissues, switching from a commensal to a causative agent of various infections such as otitis media (OM), pneumonia, and meningitis (1, 2). Although the widespread use of pneumococcal vaccines that target the polysaccharide capsule has been successful in eliciting protection against invasive pneumococcal disease (IPD), observations of serotype replacement and the increased isolation of nonencapsulated Streptococcus pneumoniae (NESp) have been reported (3, 4). NESp strains are a subgroup of pneumococci that lack a capsule and are thus categorized differently from encapsulated strains, which are grouped into serotypes determined by serological reactions between capsule and antibodies. NESp strains are placed into two groups depending on the gene content found in the capsular polysaccharide synthesis (*cps*) locus, which is the area between the highly conserved *dexB* and *aliA* genes (5). Group I NESp strains possess *cps* genes, but as a result of genetic mutation, they do not produce functional capsule polysaccharides (5). Group II NESp strains do not possess the traditional *cps* genes but instead possess genes such as *pspK*, *aliC*, and *aliD* in their place (6).

Group II NESp strains are frequently isolated from the nasopharynx of asymptomatic carriers, and although NESp strains are conventionally considered nonvirulent, group II NESp strains have been isolated from IPD cases (1, 5–7). In a U.S. study of IPD isolates, 0.61% were found to be nontypeable (5). Further examination of NESp IPD isolates revealed that 82% of strains belonged to group II NESp containing the genes *aliC* and *aliD*, designated null capsule clade 2 (NCC2) (5, 8–10). Experiments utilizing NCC2 NESp strains in animal models of infection have shown increased rates of colonization and enhanced virulence during OM and pneumonia infections in comparison to their isogenic mutants that lack the *aliC* and *aliD* genes (11). It is unknown how NESp colonizes and causes disease while lacking a capsule, an important virulence factor that possesses antiphagocytic properties and reduces clearance by complement (12, 13). However, since AliC and AliD are expressed in the majority of NESp strains that have been isolated globally from IPD cases, AliC and AliD may possess certain properties that provide a selective advantage for NESp (11). Interestingly, *aliD* is found in the *cps* locus of encapsulated strains of serotypes 25A, 25F, and 38 and also in other streptococcal species (8, 14).

AliC and AliD are substrate binding proteins that bind oligopeptides and deliver them to the Ami permease system encoded by the pneumococcal *amiACDEF* operon (15). The Ami permease system is part of the ATP binding cassette (ABC) transporter family and includes the transmembrane proteins AmiC and AmiD, the cytosolic ATPases AmiE and AmiF, and the cell membrane-anchored lipoprotein AmiA that binds and concentrates oligopeptides (15–17). AliC and AliD are paralogs of AmiA that bind unique albeit overlapping oligopeptide sequences in comparison to AmiA (8, 15). Since pneumococci have complex nutritional requirements, the importation of oligopeptides by the Ami permease system is important for adequate nutrition and growth. Moreover, many streptococcal species have oligopeptide transporter systems that have dual functions as signal transducers that can alter the expression of genes responsible for adhesion and several other virulence factors (18–22). AmiA has other paralogs; however, previous data have shown that NESp-specific AliC and AliD serve as indirect gene regulators that lead to the production of altered phenotypes (11, 16, 17).

Previous research demonstrated that oligopeptide transporters are important for infections by both encapsulated and nonencapsulated S. pneumoniae (11, 23). AliC and AliD are required for otitis media infections, increased survival in whole blood, and reductions in complement C3b deposition on the bacterial surface (11). It is unknown if AliC and AliD are directly responsible for virulence or if the regulation of downstream gene expression is responsible. Previous work examined genes regulated by AliC and AliD using both proteomics and transcriptomics (11). Of the genes identified to be regulated by AliC and AliD, one gene of particular interest was the choline binding protein AC gene (*cbpAC*) (11). Based on genomic analysis, the *cbpAC* gene is actually an allele of *pspA* and not a variant of pneumococcal surface protein C (PspC), as previously believed (11, 24–27). PspA is a virulence factor that reduces C3b and C-reactive protein (CRP) deposition on the cell surface and subsequent phagocytosis (28).

Pneumococcal clearance through C3b opsonization is initiated predominantly through the classical complement pathway (29). The activation of the pathway happens through C1q binding to an antigen-antibody complex or to CRP-bound bacteria and the formation of the C3 convertase (28, 30). This causes high rates of C3b deposition and leads to efficient clearance by opsonophagocytosis (30). NESp strains expressing AliC and AliD or CbpAC have been shown to inhibit C3b deposition, which provides protection from complement-mediated clearance and allows NESp to persist within the host (11).

The identification of unique pneumococcal processes that increase survival during systemic infection is an important first step for the development of novel preventative methods. This study demonstrates how modulating the expression of a single gene, *cbpAC*, inhibits the first step in complement deposition, allowing the increased survival and virulence of both encapsulated and nonencapsulated S. pneumoniae strains.

## RESULTS

### Pneumococcal gene regulation.

**FIG 1:**
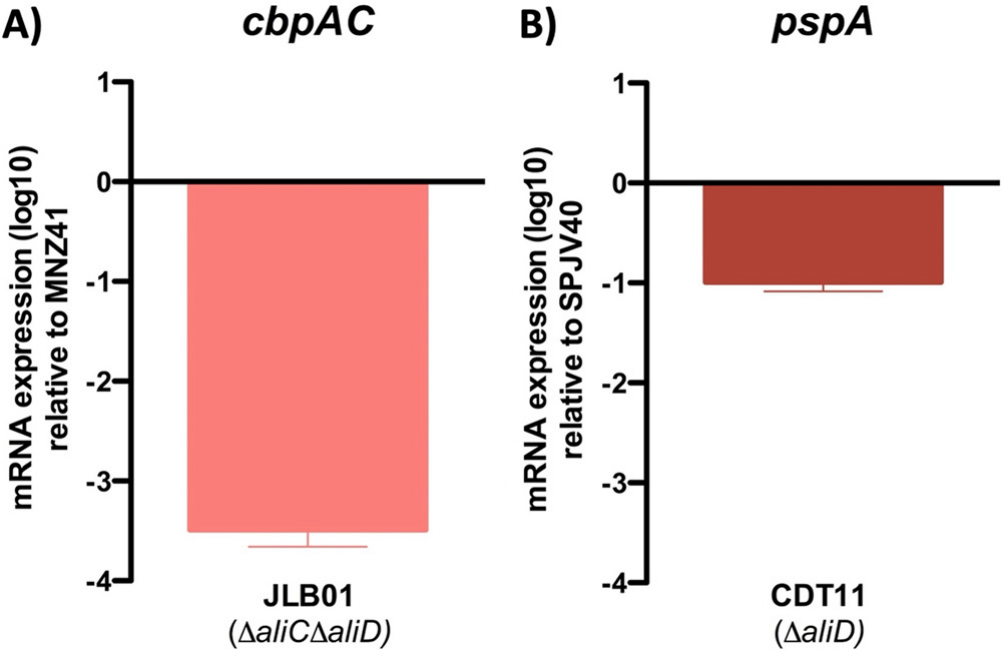
Pneumococcal gene regulation. AliC/AliD regulation of *cbpAC* in NESp and AliD regulation of *pspA* in an encapsulated serotype 38 strain were analyzed by RT-qPCR. Total RNA was extracted from MNZ41 (NESp), JLB01 (MNZ41 Δ*aliC* Δ*aliD*), SPJV40 (serotype 38), and CDT11 (SPJV40 Δ*aliD*), and cDNA was generated and utilized as a template in RT-qPCRs with primers that amplified the *cbpAC* gene (A) or the *pspA* gene (B). Average *C_T_* values were normalized to the value for the *gyrA* gene, and the fold differences were calculated using the comparative *C_T_* (2^−ΔΔ^*^CT^*) method. Panels show data from two independent biological replicates. Error bars represent the standard errors of the means.

Previous research demonstrated that AliC and AliD are required for virulence in different animal models and for survival during exposure to chinchilla whole blood and human polymorphonuclear leukocytes (11). RNA sequencing and proteomics data have shown that upon the deletion of AliC and AliD, several genes are differentially expressed (11). The *cbpAC* gene has been identified as one of the genes regulated by AliC/AliD and has been shown to reduce C3b deposition on the pneumococcal surface through an unknown mechanism (11). To determine the mechanism that reduces C3b deposition in response to CbpAC, we first wanted to verify CbpAC regulation by AliC/AliD. This was done by reverse transcription-quantitative PCR (RT-qPCR), which verified that when AliC and AliD were present, there was a >1,000-fold increase in the expression level of *cbpAC* (Fig. 1A). These results corresponded to previously published proteomics data (11). We also saw that upon the deletion of only *aliC*, *cbpAC* expression increased about 10-fold, but when only *aliD* was deleted, the change in *cbpAC* expression was not significantly different (see Fig. S2 in the supplemental material). We also investigated a unique feature possessed by encapsulated serotypes 25A, 25F, and 38: an *aliD* gene present in the *cps* locus (31). Since CbpAC is regulated by AliC and AliD, we wanted to examine the potential impact of AliD on the expression of *pspA*, which is a *cbpAC* allele. We generated an AliD mutant in the encapsulated pneumococcal serotype 38 isolate SPJV40 by allelic replacement and performed RT-qPCR on the wild-type (WT) encapsulated strain and the AliD mutant strain CDT11. When *pspA* expression in the wild-type SPJV40 (serotype 38) strain was compared to that of the isogenic AliD mutant (CDT11), we saw a 10-fold reduction in expression (Fig. 1B).

10.1128/mbio.03325-22.3 FIG S2 Variations in C3b deposition and pneumococcal gene regulation. MNZ41 (NESp) and JLB02 (MNZ41 Δ*aliC*) were incubated with 20% NHS, followed by the detection of C3b binding by flow cytometry, and AliC/AliD regulation of *cbpAC* in NESp was analyzed by RT-qPCR. (A) Flow cytometry histogram indicating no shift in C3b deposition upon *aliC* deletion (31.82%) compared to WT MNZ41 (31.08%). (B) The mean fluorescence intensity of C3b deposition on the population over the nonstained level indicated no significant difference in total C3b deposition between the two populations. (C) Total RNA was extracted from MNZ41 (WT NESp), JLB02 (MNZ41 Δ*aliC*), and JLB04 (MNZ41 Δ*aliD*), and cDNA was generated and utilized as a template in RT-qPCRs with primers that amplified the *cbpAC* gene. Average *C_T_* values were normalized to the value for the *gyrA* gene, and the fold differences were calculated using the comparative *C_T_* (2^−ΔΔ^*^CT^*) method (45). Data are from two independent biological replicates. Error bars represent the standard errors of the means. Download FIG S2, TIF file, 1.2 MB. Copyright © 2023 Thompson et al. 2023 Thompson et al. https://creativecommons.org/licenses/by/4.0/ This content is distributed under the terms of the Creative Commons Attribution 4.0 International license.

### Variations in CRP deposition.

**FIG 2:**
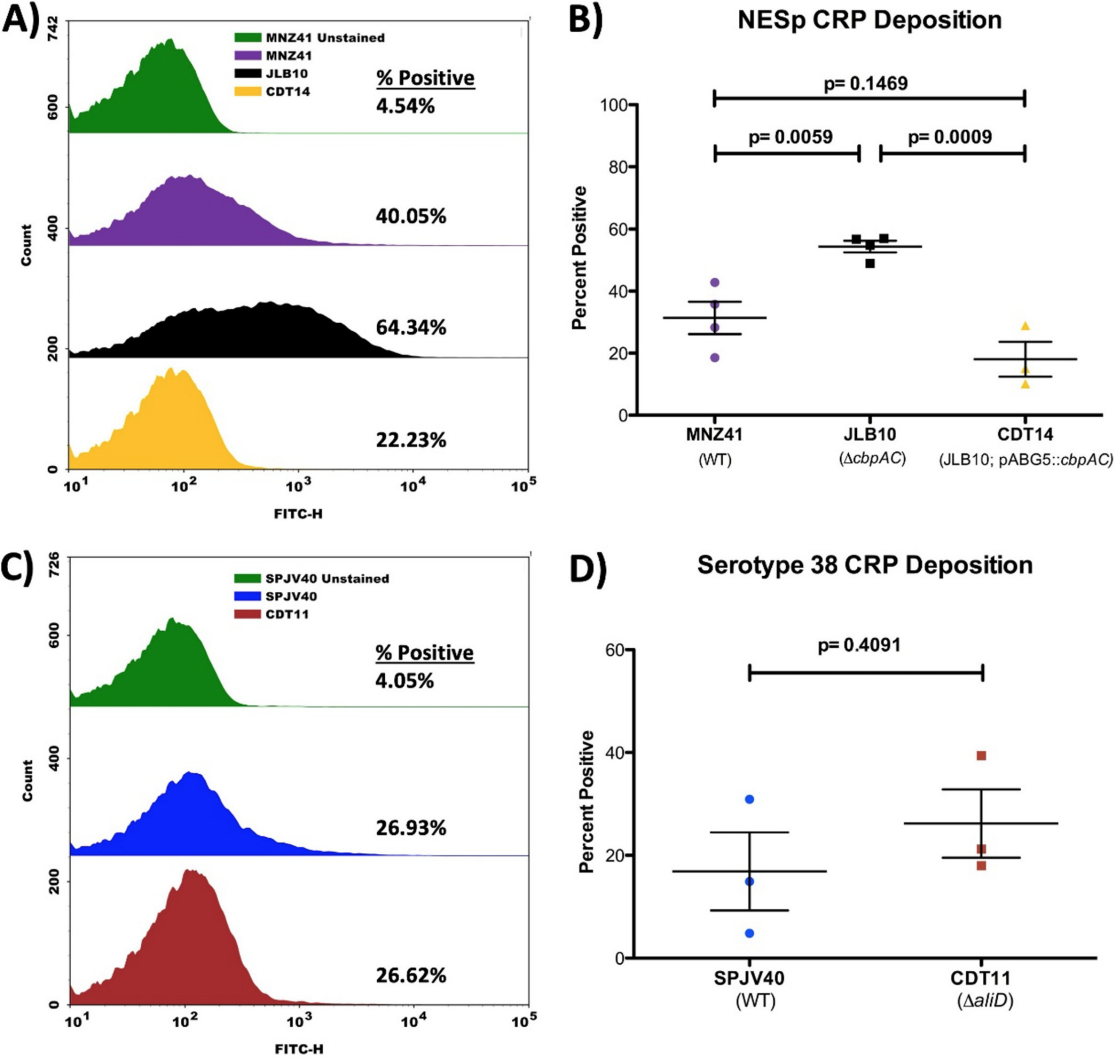
Variations in CRP deposition. MNZ41 (NESp), JLB10 (MNZ41 Δ*cbpAC*), CDT14 (JLB10/pABG5::*cbpAC*), SPJV40 (serotype 38), and CDT11 (SPJV40 Δ*aliD*) were incubated with 50% NHS following the detection of CRP deposition by flow cytometry. (A) Flow cytometry histogram indicating a shift in CRP deposition upon *cbpAC* deletion (64.34%) compared to WT MNZ41 (40.05%) and the *cbpAC*-complemented strain CDT14 (22.23%). (B) The mean fluorescence intensity of CRP deposition on the population over the nonstained level indicated no significant difference in total CRP deposition among the three populations. (C) Flow cytometry histogram indicating no shift in CRP deposition upon *aliD* deletion (26.62%) compared to WT SPJV40 (26.93%). (D) The mean fluorescence intensity of CRP deposition on the population over the nonstained level indicated no significant difference in total CRP deposition between the two populations. Histograms are representative of results from three independent experiments, and percent positivity values are the averages for all experiments performed. Error bars represent standard errors of the means.

Previous research indicated that JLB01 (MNZ41 Δ*aliC* Δ*aliD*) binds C3b at higher rates than the WT and that the presence of CbpAC plays a role in this function (11). We have now shown that AliC and AliD regulate the expression of CbpAC, a PspA homolog, a surface protein that has previously been shown to reduce CRP deposition (28). Therefore, we wanted to determine the role of CbpAC in CRP deposition, which is utilized in C3b deposition through the classical pathway, an important mechanism of pneumococcal clearance. Using flow cytometry, CRP deposition was determined in the AliC AliD double mutant strain (JLB01 [MNZ41 Δ*aliC* Δ*aliD*]), the CbpAC deletion mutant of NESp MNZ41 (JLB10 [MNZ41 Δ*cbpAC*]), and its complemented strain CDT14 (JLB10/pABG5::*cbpAC*). In NESp strain MNZ41, the deletion of AliC/AliD caused an increase in CRP deposition (38.03%) compared to WT MNZ41 (31.29%) and consistently showed higher levels of deposition in multiple experiments (Fig. S3). The deletion of CbpAC significantly increased CRP deposition (*P* = 0.0059) in comparison to WT MNZ41, and the complemented strain CDT14 had binding restored to WT levels (Fig. 2A and B).

10.1128/mbio.03325-22.4 FIG S3 Variations in CRP deposition. MNZ41 (NESp) and JLB01 (MNZ41 Δ*aliC* Δ*aliD*) were incubated with 50% NHS following the detection of CRP deposition by flow cytometry. (A) Flow cytometry histogram indicating a slight shift in CRP deposition upon *aliC* and/or *aliD* deletion (38.03%) compared to WT MNZ41 (31.29%). (B) The mean fluorescence intensity of CRP deposition on the population over the nonstained level indicated no significant difference in total CRP deposition between the two populations. Histograms are representative of results from a minimum of three independent experiments, and percent positivity values are the averages for all experiments performed. Error bars represent standard errors of the means. Download FIG S3, TIF file, 1.1 MB. Copyright © 2023 Thompson et al. 2023 Thompson et al. https://creativecommons.org/licenses/by/4.0/ This content is distributed under the terms of the Creative Commons Attribution 4.0 International license.

Since we have demonstrated that *pspA* expression is reduced in serotype 38 strain SPJV40 upon the deletion of *aliD*, we also wanted to assess CRP deposition on the pneumococcal surface. Using flow cytometry, CRP deposition was determined in the WT and the AliD deletion mutant of SPJV40 (CDT11 [SPJV40 Δ*aliD*]). In serotype 38 WT strain SPJV40, CRP was found to bind at 26.93%, compared to 26.62% binding for the isogenic *aliD* deletion mutant (Fig. 2C). The *aliD* deletion mutant of SPJV40 did not show an increase in CRP deposition across multiple experiments compared to the WT strain (Fig. 2D), which could be a result of protection against CRP deposition provided by the capsule.

### Variations in C3b deposition.

**FIG 3:**
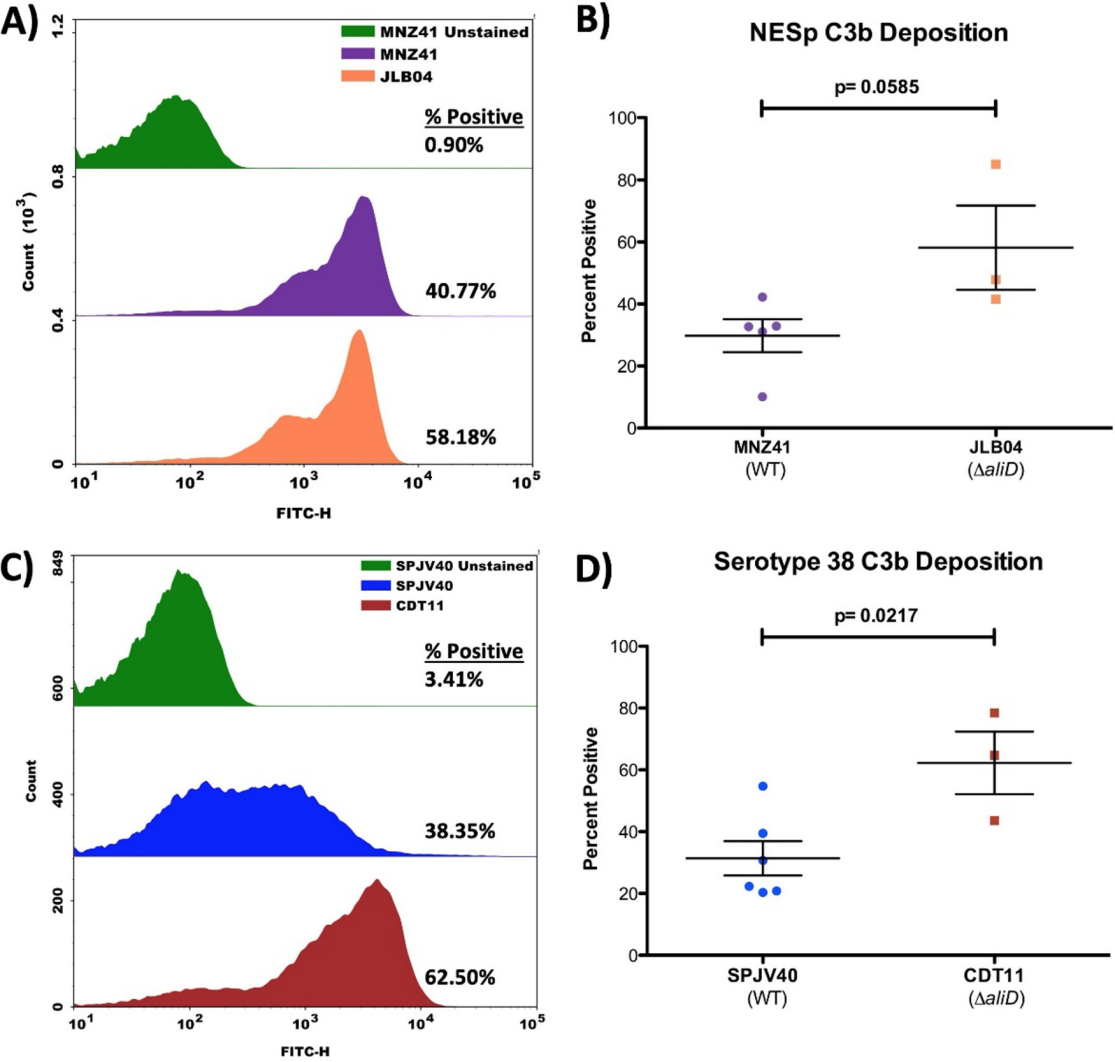
Variations in C3b deposition. MNZ41 (NESp), JLB04 (MNZ41 Δ*aliD*), SPJV40 (serotype 38), and CDT11 (SPJV40 Δ*aliD*) were incubated with 20% NHS, followed by the detection of C3b binding by flow cytometry. (A) Flow cytometry histogram indicating a shift in C3b deposition upon *aliD* deletion (58.18%) compared to WT MNZ41 (40.77%). (B) The mean fluorescence intensity of C3b deposition on the population over the nonstained level indicated no significant difference in total C3b deposition between the two populations. (C) Flow cytometry histogram indicating a significant shift in C3b deposition upon *aliD* deletion (62.50%) compared to encapsulated strain SPJV40 (38.35%). (D) The mean fluorescence intensity of C3b deposition on the population over the nonstained level indicated no significant difference in total C3b deposition between the two populations. Histograms are representative of results from three independent experiments, and percent positivity values are the averages for all experiments performed. Error bars represent standard errors of the means.

CbpAC was previously shown to affect C3b deposition, and the deletion of AliC/AliD reduces the amount of CbpAC present (11). It was also previously shown that PspA of D39 (serotype 2) inhibits the complement cascade and reduces C3b and CRP deposition on the cell surface (28). Therefore, we wanted to assess C3b deposition on the bacterial surface as a function of AliD. Using flow cytometry, C3b deposition was determined in the AliD deletion mutants of NESp MNZ41 (JLB04 [MNZ41 Δ*aliD*]) and serotype 38 SPJV40 (CDT11 [SPJV40 Δ*aliD*]). In NESp strain MNZ41, C3b was found to bind 40.77% of cells, compared to 58.18% binding in the isogenic *aliD* deletion mutant (Fig. 3A). The increase in C3b deposition was consistent across multiple experiments but was not significant (*P* = 0.0585) (Fig. 3B). While the AliD deletion is sufficient to slightly increase C3b deposition in NESp, a greater effect was previously observed when both AliC and AliD were deleted (11). Therefore, other experiments focused on the JLB01 double mutant of NESp.

Next, we wanted to examine C3b deposition on the encapsulated pneumococcus and its AliD mutant. In serotype 38 strain SPJV40, the deletion of AliD caused a significant increase in C3b deposition (62.50%) in comparison to that of WT SPJV40 (38.35%) (*P* = 0.0217) (Fig. 3C and D). To verify that the deletion of *aliD* from the *cps* locus did not disrupt capsule production, we performed negative capsule staining with nigrosin and saw no difference in capsule production (Text S1, Fig. S4). Therefore, AliD expression in encapsulated strains is sufficient to significantly reduce C3b deposition on the bacterial surface.

10.1128/mbio.03325-22.5 FIG S4 Capsule expression in an encapsulated serotype 38 AliD mutant. Encapsulated serotype 38 WT strain SPJV40 and CDT11 (SPJV40 Δ*aliD*) were stained with nigrosin and crystal violet and visualized under a light microscope to verify that the deletion of *aliD* from the *cps* locus in SPJV40 did not disrupt capsule production. (A and B) Microscopic analysis determined the presence of capsule in both WT SPJV40 (A) and CDT11 (SPJV40 Δ*aliD*) (B). (C) Analysis of capsule production determined a nonsignificant difference (*P* value of 0.606) in capsule layer thickness between WT strain SPJV40 and CDT11 (SPJV40 Δ*aliD*). Captured images were analyzed using ImageJ software. Error bars represent standard errors of the means. Download FIG S4, TIF file, 2.9 MB. Copyright © 2023 Thompson et al. 2023 Thompson et al. https://creativecommons.org/licenses/by/4.0/ This content is distributed under the terms of the Creative Commons Attribution 4.0 International license.

### Complement component binding.

Our flow cytometry analysis showed that the presence of AliD in NESp and encapsulated serotype 38 strains reduces C3b deposition and that the increased expression of CbpAC/PspA is important for this. It is unknown how AliD regulation of CbpAC and PspA reduces complement deposition. For this reason, our next step was to determine which complement pathway—classical, alternative, or lectin—is inhibited by CbpAC and results in less C3b deposition on the pneumococcal surface. To determine variations in the efficiencies of binding of host-derived factors to WT MNZ41 and JLB01 (Δ*aliC* Δ*aliD*), both strains were incubated with human plasma, followed by liquid chromatography-tandem mass spectrometry (LC-MS/MS) to identify bound proteins. There were 48 identified human plasma proteins that bound to the WT MNZ41 and 44 that bound to the AliC/AliD mutant strain JLB01 (Table 1; Table S1). Analysis of the spectral count, the total number of MS/MS spectra that matched to an assigned protein, determined that there were 517 total spectral counts for MNZ41 and 700 for the AliC/AliD mutant. Out of those total spectral counts, our results revealed that significantly higher concentrations of C1q (*P* = 0.003) and CRP (*P* = 0.038) were present on the surface of the AliC/AliD mutant than on the WT. C1q and CRP are involved in the activation of the classical pathway of the complement system. Therefore, the reduction in C3b deposition occurs by inhibiting the first step in the classical complement pathway.

**TABLE 1:** MS/MS spectral count comparison of MNZ41 and JLB01^*a*^

Sample	Total no. of identified proteins	Total no. of spectra	Total no. of peptides		No. of spectra	
			Human C1q	Human CRP	Human C1q	Human CRP
MNZ41 (WT)	48	517	1	1	6	7
JLB01 (Δ*aliC* Δ*aliD*)	44	700	4	2	21	17

a The spectral count, a semiquantitative index, is the total number of MS/MS spectra that are matched to an assigned protein. The *P* values were 0.003 for the human C1q spectral count and 0.038 for the human CRP spectral count.

10.1128/mbio.03325-22.9 TABLE S1 Spectral count comparison of proteins identified from the WT and mutant samples. Download Table S1, XLSX file, 0.02 MB. Copyright © 2023 Thompson et al. 2023 Thompson et al. https://creativecommons.org/licenses/by/4.0/ This content is distributed under the terms of the Creative Commons Attribution 4.0 International license.

### Murine survival studies.

**FIG 4:**
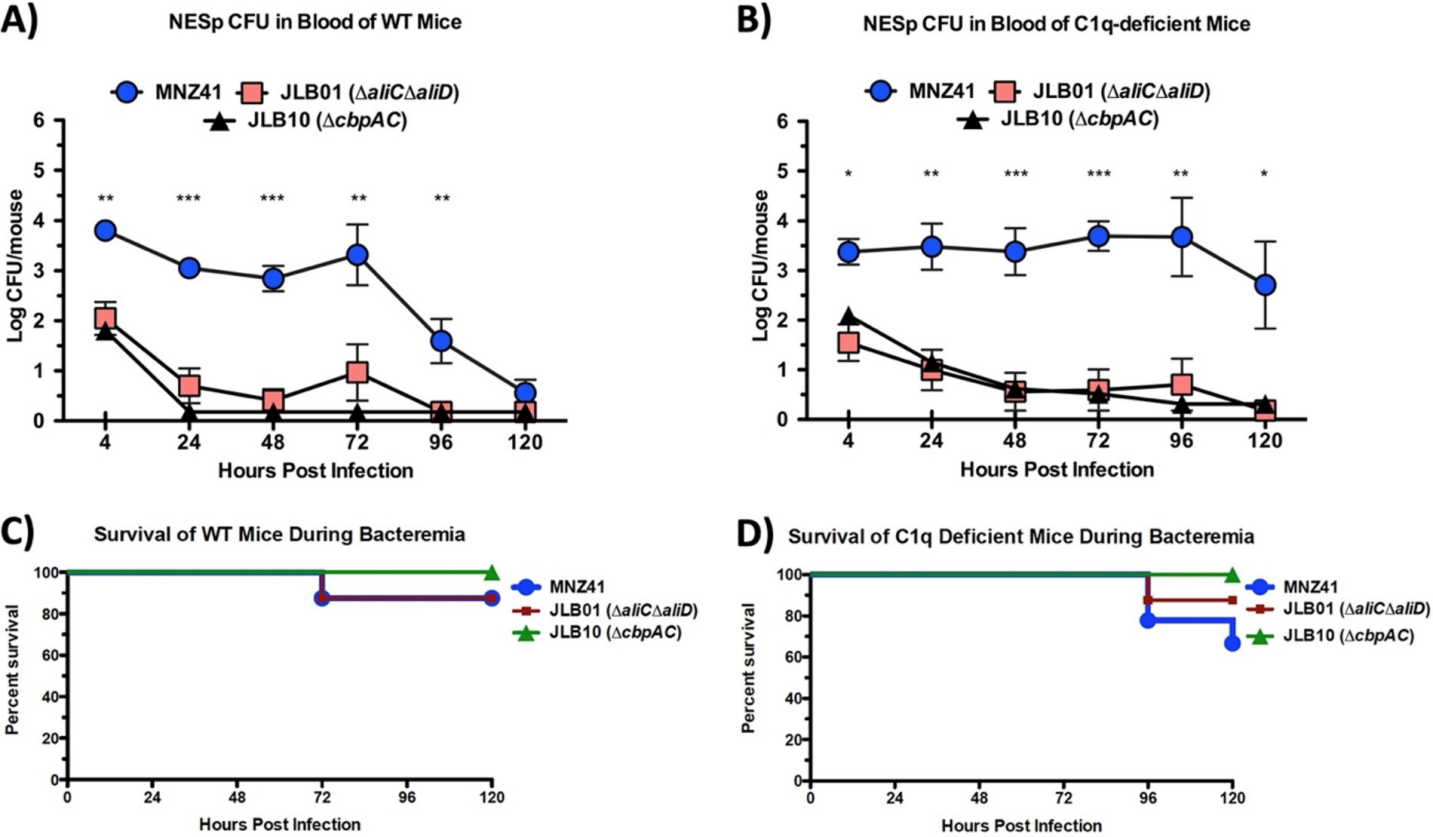
Systemic survival of NESp in WT and C1q-deficient mice and analysis of murine survival following bacteremia. C57BL/6 mice were challenged intraperitoneally (i.p.) with MNZ41 and the isogenic mutants to determine bacterial systemic survival and murine mortality. (A and B) Quantifications of the systemic survival of MNZ41 (WT), JLB01 (Δ*aliC* Δ*aliD*), and JLB10 (Δ*cbpAC*) at the indicated time points after i.p. injection of WT mice (A) and C1q-deficient mice (B). (C and D) Percentages of survival of WT mice (C) and C1q-deficient mice (D) following bacteremia were calculated at the indicated time points after bacterial injection. Clearance of MNZ41 from the blood is dependent on C1q, whereas the isogenic mutant strains JLB01 (Δ*aliC* Δ*aliD*) and JLB10 (Δ*cbpAC*) are cleared independently of the C1q status. A minimum of 4 mice per strain were used for each time point in two independent experiments. Significance indicates MNZ41 variation from both mutant strains JLB01 and JLB10. *, *P* < 0.05; **, *P* < 0.01; ***, *P* < 0.001.

Our data have shown that the presence of AliD reduces C3b deposition in encapsulated strains and increases PspA expression. In NESp, C3b deposition and CbpAC regulation are altered when AliD is present, but the phenotype is more strongly observed when both AliC and AliD are present. Moreover, the activation of the classical complement pathway is inhibited by AliC and AliD. We next wanted to demonstrate that by regulating the expression of CbpAC by AliC and AliD, NESp strains are better able to survive clearance during invasive infection by inhibiting the activation of the classical complement pathway. Therefore, we utilized a murine model with WT and C1q-deficient mice in a C57BL/6 background to examine the role of complement during *in vivo* NESp infection. WT and C1q-deficient mice were challenged intraperitoneally (i.p.) with WT MNZ41, JLB01 (Δ*aliC* Δ*aliD*), and JLB10 (Δ*cbpAC*) to determine virulence and bacterial systemic survival. Upon infection, WT MNZ41 maintained a consistent bacterial burden in the blood of WT mice for 3 days before a reduction in bacterial levels was seen. In contrast, the bacterial burdens of JLB01 (Δ*aliC* Δ*aliD*) and JLB10 (Δ*cbpAC*) in the blood were barely detectable by 24 h postinfection in WT mice (Fig. 4A; Fig. S5). At all time points besides 120 h postinfection, WT MNZ41 bacteria were recovered at significantly higher levels than both JLB01 and JLB10. JLB01 and JLB10 were also cleared from C1q-deficient mice as rapidly as in WT mice, but in contrast, MNZ41 continued to persist after 5 days, with no significant reduction in the bacterial load (Fig. 4B; Fig. S5). At both 96 and 120 h postinfection, MNZ41 was recovered from the blood at significantly higher levels from C1q-deficient mice than from WT mice (*P* < 0.05) (Fig. S5A). Furthermore, murine mortality following bacteremia was also determined. Compared to the 12.5% mortality rate in WT mice (Fig. 4C), there was a 33% mortality rate in C1q-deficient mice challenged with MNZ41 (Fig. 4D). However, no significant difference in survival between WT and C1q-deficient mice was observed (Fig. S6). Our results indicate that the clearance of MNZ41 from the blood is dependent upon C1q, whereas the isogenic mutants JLB01 (Δ*aliC* Δ*aliD*) and JLB10 (Δ*cbpAC*) are cleared independently of the C1q status.

10.1128/mbio.03325-22.6 FIG S5 Systemic survival of NESp in WT and C1q-deficient mice following bacteremia. C57BL/6 mice were challenged intraperitoneally (i.p.) with MNZ41 and the isogenic mutants to determine bacterial systemic survival. The rates of systemic survival of MNZ41 (WT) (A), JLB01 (Δ*aliC* Δ*aliD*) (B), and JLB10 (Δ*cbpAC*) (C) are shown at the indicated time points after i.p. injection of WT mice (indicated by closed circles) and C1q-deficient mice (indicated by open circles). Clearance of MNZ41 from the blood is dependent on C1q, whereas the isogenic mutant strains JLB01 (Δ*aliC* Δ*aliD*) and JLB10 (Δ*cbpAC*) are cleared independently of the C1q status. A minimum of 4 mice per strain were used for each time point in two independent experiments (*n* = 8). Significance indicates variation between WT mice and C1q-deficient mice. An asterisk denotes significance (*, *P* < 0.05; **, *P* < 0.01; ***, *P* < 0.001). Download FIG S5, TIF file, 2.9 MB. Copyright © 2023 Thompson et al. 2023 Thompson et al. https://creativecommons.org/licenses/by/4.0/ This content is distributed under the terms of the Creative Commons Attribution 4.0 International license.

10.1128/mbio.03325-22.7 FIG S6 Analysis of murine survival following bacteremia. C57BL/6 mice were challenged intraperitoneally (i.p.) with MNZ41 to determine murine mortality. The percentages of survival of WT mice (indicated by closed circles) and C1q-deficient mice (indicated by open circles) following bacteremia were calculated at the indicated time points after bacterial injection. A minimum of 4 mice per strain were used for each time point in two independent experiments. No significant difference in survival (Mantel-Cox log rank test) was observed between WT and C1q-deficient mice. Download FIG S6, TIF file, 1.2 MB. Copyright © 2023 Thompson et al. 2023 Thompson et al. https://creativecommons.org/licenses/by/4.0/ This content is distributed under the terms of the Creative Commons Attribution 4.0 International license.

### Opsonophagocytosis.

**FIG 5:**
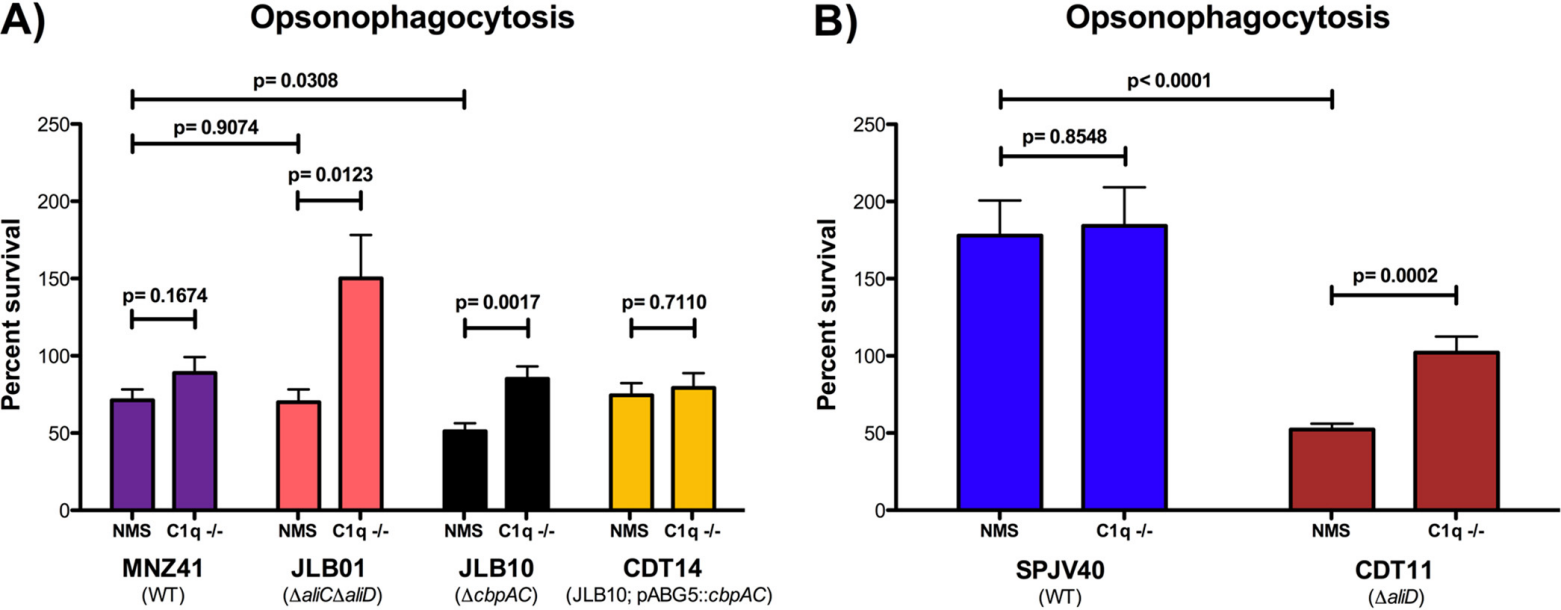
Opsonophagocytosis following preincubation with normal or C1q-deficient mouse serum. Bacterial strains were incubated with 3% mouse serum, normal or C1q deficient, followed by incubation with HL-60 cells differentiated into neutrophils. (A) The percentages of survival after opsonophagocytosis of strains MNZ41 (NESp), JLB01 (MNZ41 Δ*aliC* Δ*aliD*), JLB10 (MNZ41 Δ*cbpAC*), and CDT14 (JLB10/pABG5::*cbpAC*) were calculated by comparing the CFU of strains incubated with and those incubated without neutrophils. Significant increases in bacterial survival were observed for strains JLB01 and JLB10 incubated with C1q-deficient serum compared to NMS. Complementation with *cpbAC* in CDT14 restored survival rates to wild-type levels. (B) The percentages of survival after opsonophagocytosis of strains SPJV40 (serotype 38) and CDT11 (SPJV40 Δ*aliD*) were calculated by comparing the CFU of strains incubated with and those incubated without neutrophils. Bacterial survival was significantly decreased in CDT11 incubated with NMS compared to C1q-deficient serum.

We have shown that through the regulation of *cbpAC* expression by AliD, there are altered rates of CRP and C3b deposition. Murine studies demonstrate the rapid clearance of mutants compared to WT bacteria in both WT and C1q-deficient mice. Due to the unexpected clearance of mutant strains in C1q-deficient mice, opsonophagocytosis assays using serum from normal or C1q-deficient mice as the opsonin source were performed. Upon incubation with either serum source, MNZ41 survival upon opsonophagocytosis did not differ, but the survival of JLB01 (Δ*aliC* Δ*aliD*) was significantly increased in C1q-deficient serum compared to normal mouse serum (NMS) (*P* = 0.0123) (Fig. 5A). The survival of JLB10 (Δ*cbpAC*) was also significantly increased in C1q-deficient serum compared to NMS (*P* = 0.0017), and the restoration of the wild-type phenotype was observed when *cbpAC* was complemented (Fig. 5A). The independent deletion of either *aliC* or *aliD* had no effect on opsonophagocytosis survival in either serum source (Fig. S7). A comparison of MNZ41 to JLB01 (Δ*aliC* Δ*aliD*) or JLB10 (Δ*cbpAC*) incubated in NMS showed a significant decrease in the survival of JLB10 compared to the WT (*P* = 0.031) but not when JLB01 was compared to the WT. Opsonophagocytosis was also tested in encapsulated strain SPJV40 and the isogenic *aliD* mutant strain CDT11. There was no difference in the opsonophagocytosis of SPJV40 when preincubated with either serum source, but there was a significant increase in the survival of CDT11 when incubated with C1q-deficient serum (*P* = 0.0002) (Fig. 5B). There was also a significant decrease in the survival of CDT11 (Δ*aliD*) incubated in NMS compared to WT SPJV40 (*P* < 0.0005).

10.1128/mbio.03325-22.8 FIG S7 Opsonophagocytosis following preincubation with normal or C1q-deficient mouse serum. Bacterial strains were incubated with 3% mouse serum, normal or C1q deficient, followed by incubation with HL-60 cells differentiated into neutrophils. The percent survival after opsonophagocytosis of strains MNZ41 (NESp), JLB02 (MNZ41 Δ*aliC*), and JLB04 (MNZ41 Δ*aliD*) was calculated by comparing the CFU of strains incubated with and those incubated without neutrophil-like cells. No significant increases in bacterial survival were observed for strains incubated with C1q-deficient serum compared to NMS. Download FIG S7, TIF file, 1.6 MB. Copyright © 2023 Thompson et al. 2023 Thompson et al. https://creativecommons.org/licenses/by/4.0/ This content is distributed under the terms of the Creative Commons Attribution 4.0 International license.

## DISCUSSION

**FIG 6:**
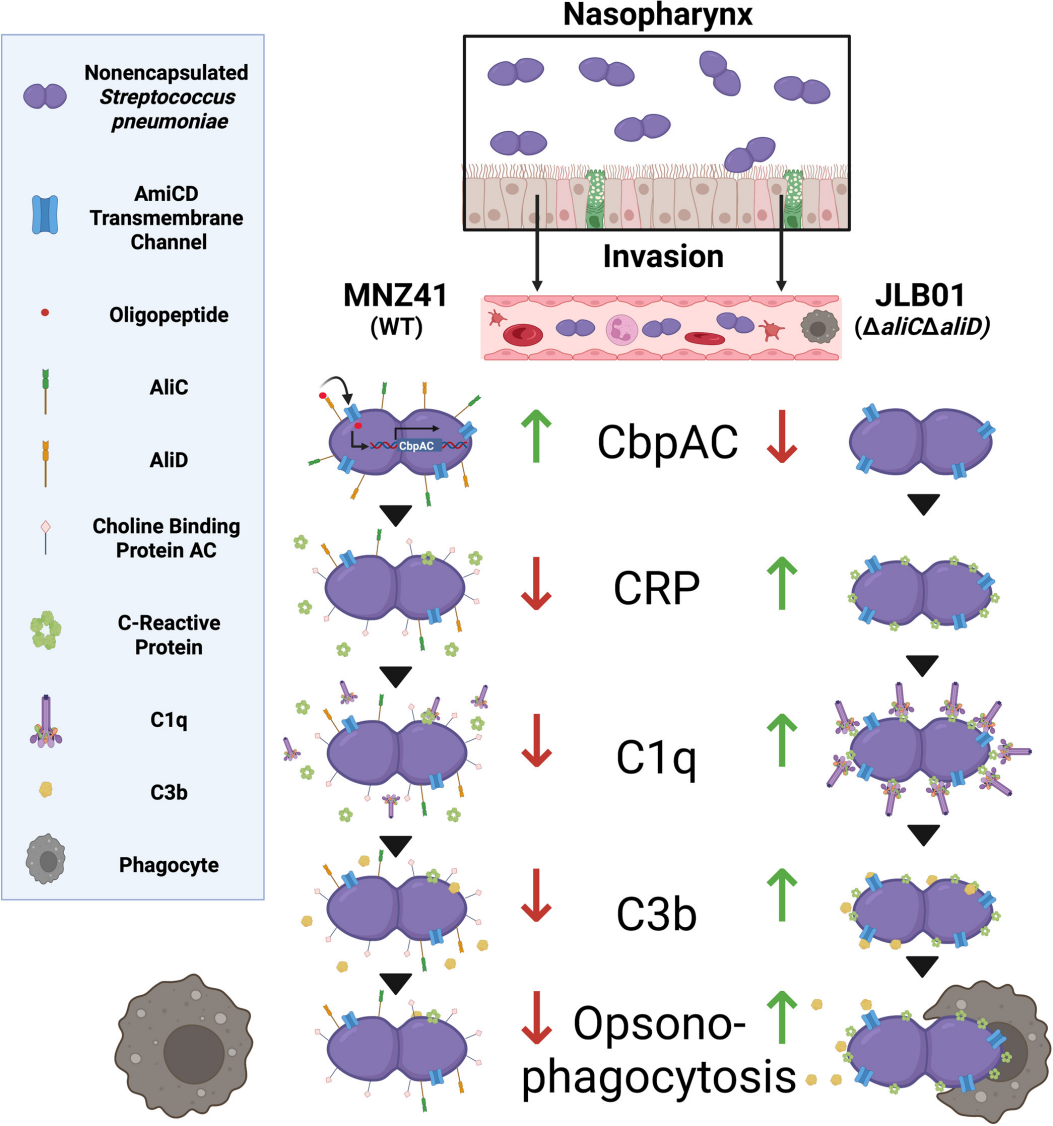
Model of the AliC/AliD regulatory pathway of CbpAC. The inhibition pathway of AliC/AliD-regulated CbpAC that results in decreased C3b deposition and increased systemic survival is highlighted. During invasive disease, NESp strains expressing AliC and AliD are able to regulate the expression of CbpAC. Increased CbpAC expression leads to decreased binding of CRP to the phosphocholine on the pneumococcal cell wall. As CRP is known to activate the classical pathway of the complement system, a reduction in CRP deposition leads to a decrease in classical complement activity: reduced C1q deposition and subsequent C3b deposition. Without C3b deposition on the surface of the cell, there is a decrease in opsonophagocytosis, ultimately leading to increased NESp survival during systemic infections. (Figure created using BioRender [https://biorender.com/].)

This study assesses the mechanism of antiphagocytic activity upon the expression of AliC or AliD. We found that through the regulation of CbpAC in NESp, there is reduced C-reactive protein binding, which in turn reduces complement deposition and increases virulence during systemic infections (Fig. 6). Encapsulated strains that encode AliD also have reduced complement deposition and increased *pspA* expression. This is a novel mechanism for the regulation of one of the major virulence factors of the pneumococcus and the first demonstration of how NESp survives in the blood and causes systemic infections. An increased understanding of how NESp strains are able to cause disease is necessary as vaccines targeting a wider range of capsule serotypes are being developed and introduced into the population.

Previous work demonstrated the importance of AliC, AliD, and CbpAC in reducing C3b deposition and decreasing phagocytosis (11). It is unknown whether AliC and AliD work independently to regulate CbpAC expression or if both proteins are necessary for the phenotypic changes observed. Therefore, our first step was to determine how changes in C3b deposition are affected in independent mutants, JLB02 (Δ*aliC*) and JLB04 (Δ*aliD*) (Fig. 3; see also Fig. S2 in the supplemental material). Interestingly, the deletion of either AliC or AliD was not sufficient to increase C3b deposition to the levels observed when both proteins were deleted. A unique feature of group II NESp strains is their division into three separate groups: NCC1 (*pspK*+), NCC2 (*aliC*+ *aliD*+), and NCC3 (*aliD*+). To date, no strain of NESp containing only the *aliC* gene has been isolated. We therefore assumed that AliD would be sufficient to reduce C3b deposition, and while it was responsible for some of the phenotypic changes observed, the deletion of both proteins was required for the significant increase in C3b deposition observed previously (11).

Analyses of the *cps* locus of encapsulated strains indicate that serotypes 25A, 25F, and 38 all possess the *aliD* gene within the *cps* locus (31). These three serotypes are not included in any of the available pneumococcal vaccines but still cause systemic disease (31–33). We wanted to determine if AliD in an encapsulated strain would provide the same protection during systemic infection. We examined the ability of C3b to be deposited in a wild-type encapsulated strain and an isogenic *aliD* mutant and found that significantly less C3b was deposited on the surface of the wild-type strain. Typically, the presence of the pneumococcal capsule is necessary to establish invasive disease as it functions to assist in colonization, immune evasion, and the prevention of clearance by the host mucosa, but this is not always the case (3, 9, 12, 13, 34, 35). A recent publication reported the first documented case of infectious endocarditis caused by a group I (capsule mutant) NESp strain. Whole-genome sequencing revealed that the endocarditis isolate was a serotype 38 strain with a premature stop codon for a gene that is necessary for functional polysaccharide expression found downstream of the *aliD* gene (36). That group confirmed that the patient in whom the strain was isolated had no underlying immunodeficiency and suggested that the explanation for the rare infectious endocarditis case was increased bacterial fitness in the blood. Increased systemic survival may result from the presence of AliD in this endocarditis isolate, and no current vaccine would be effective against these strains even if this specific serotype was included in the vaccine.

It has been hypothesized that the regulation of *cbpAC* or *pspA* expression by AliC and AliD alters the bacterial virulence previously observed in both nonencapsulated and encapsulated strains (11). We first validated the regulation of CbpAC and PspA by AliC and AliD in strains that naturally express these genes, MNZ41 (NESp) and SPJV40 (serotype 38). Using RT-qPCR, we show that AliC and AliD regulated the expression of CbpAC (Fig. 1A). We also show that PspA levels in encapsulated strain SPJV40 were significantly reduced when *aliD* was deleted (Fig. 1B). These data are the first to show the regulation of *pspA*, an important and diverse virulence factor, by an oligopeptide transporter. Since *pspA* or one of its alleles is found in all pneumococcal strains, further research into the mechanism of AliD regulation of PspA expression is needed to increase our understanding of nonencapsulated and encapsulated pneumococci (37).

Previous research has shown the importance of AliC/AliD in the regulation of genes such as *cbpAC*, which aids in immune evasion by reducing C3b deposition on the pneumococcal surface, and our data support these findings (Fig. 3) (11). However, prior to our study, the stepwise inhibitory pathway of CbpAC was unknown. We wanted to examine the mechanism that causes the observed reduction in C3b deposition through AliC and AliD regulation of CbpAC expression. To determine the complement effectors involved in NESp clearance, we performed plasma binding studies, which showed that C1q and CRP deposition is increased in the AliC/AliD mutant compared to WT NESp. The classical complement pathway is initiated through C1q deposition and is targeted to the bacterial surface through antibodies or CRP binding to bacteria. By reducing the ability of CRP to bind to the bacterial surface, the complement cascade can be inhibited, leading to better survival during systemic infection. The deletion of CbpAC increased CRP binding to the bacterial surface, and following complementation, CRP deposition was reduced (Fig. 2A). The 1,000-fold decrease in CbpAC expression following AliC and AliD deletion led to a significant increase in CRP binding to the bacterial surface, as determined by plasma binding assays (Table 1). Flow cytometry indicated a slight increase in CRP binding to the double mutant strain JLB01 in comparison to the dramatic increase upon the deletion of CbpAC (Fig. 2A; Fig. S3). This can be due to a low level of CbpAC expression still being present in the JLB01 mutant. Through increasing CbpAC expression by AliC and AliD, CRP deposition is further reduced. Flow cytometry was also used to examine CRP deposition on encapsulated strain SPJV40 and its isogenic *aliD* mutant, but CRP deposition was not altered despite increases in C3b deposition. Through increasing CbpAC expression by AliC and AliD, inhibition of complement deposition occurs by reducing classical pathway activation. This theory was further tested using a murine model with C1q-deficient mice. Our data from murine studies showed that C1q-deficient mice clear WT NESp less efficiently than do WT mice, but AliC/AliD mutant clearance is independent of C1q (Fig. S5). This indicates that other mechanisms, such as the alternative complement pathway, are able to compensate for this C1q deficiency when CbpAC is absent or expressed at significantly lower levels than the wild type.

An interesting observation in our murine studies was that the deletion of AliC and AliD still caused mortality in a single mouse, in both wild-type and C1q-deficient mice (Fig. 4C and D). Low levels of CbpAC are still expressed in this double mutant, providing limited protection from the classical pathway, which could explain this observation. This is further strengthened by the survival of all mice, WT and C1q deficient, challenged with CbpAC mutant strain JLB10 (Fig. 4C and D). Another interesting observation is that WT mice infected with WT NESp maintain bacterial levels 3 days after infection before clearance starts to occur at 4 days postinfection, suggesting that this clearance could be a result of a primary immune response against the bacteria (Fig. 4A; Fig. S5A). Both JLB01 (Δ*aliC* Δ*aliD*) and JLB10 (Δ*cbpAC*) are rapidly cleared in C1q-deficient mice, which was surprising due to the inhibition of the classical complement pathway. Opsonophagocytosis assays were performed to examine this more closely after incubation with either normal mouse serum or C1q-deficient serum. As expected, there was greater survival of mutant strains incubated in C1q-deficient serum. This indicates that the lack of C1q reduces opsonization and increases survival when bacteria are incubated with neutrophil-like cells, but other *in vivo* mechanisms must account for the rapid reduction in the bacterial burden in C1q-deficient mice. Previous work has shown that CRP is able to directly bind Fc receptors on monocytes and neutrophils, promoting bacterial clearance (38, 39). Serum levels of CRP rapidly increase upon inflammation, which could be responsible for the rapid clearance observed in mouse models. Low levels of circulating CRP in serum used for opsonophagocytosis assays can account for increased survival when strains are incubated with C1q-deficient serum. Taken together, these experiments show that the systemic survival of NESp is enhanced by AliC and AliD through blocking CRP deposition and reducing C3b opsonization.

This study demonstrates that the expression of AliC and AliD provides a selective advantage for NESp, allowing survival in the blood despite the lack of a capsule. As a result, NESp strains expressing AliC and AliD have been isolated from IPD cases (11, 27). Studies show that group II NESp strains are prevalent worldwide (4 to 19% of carriage isolates) and may be increasing in prevalence due to increased pneumococcal vaccination rates (1, 40). With the introduction of newer conjugate vaccines that augment serotype coverage, there will be even more selective pressure against encapsulated strains. Because of this, there is speculation that NESp could eventually be a standard causative agent of IPD, which would add to the burden of pneumococcal disease that already exists (7). Furthermore, most studies of pneumococcal pathogenesis have focused on encapsulated strains, and thus, there is little understanding of group II NESp strains and the novel genes that they possess. Since NESp strains have high transformation efficiencies and harbor resistance to multiple drugs, there is a cumulative risk for intraspecies horizontal gene transfer to occur, which would add challenges to the current treatment strategies that we employ for the control of pneumococcal disease (41–43). Therefore, deciphering how NESp strains survive within a host and facilitate disease is a necessity that will allow the fabrication of improved, broad-spectrum treatments against pneumococcal disease, which is of importance since NESp cases are on the rise and pneumococcal vaccines are ineffective against NESp (5, 10).

In conclusion, we know that AliC and AliD contribute to NESp survival in the blood and the production of systemic disease by regulating CbpAC expression and inhibiting the classical complement pathway. Future work can examine modulators of oligopeptide transporters, like AliC and AliD, that alter gene expression required for pneumococcal survival. All encapsulated strains have alternative oligopeptide transporters as well, so the creation of a general oligopeptide transporter antagonist could aid in pneumococcal clearance during infection caused by both encapsulated and nonencapsulated strains. Altogether, the results of this work demonstrate one mechanism that NESp strains utilize for systemic survival and highlight the need for future research on oligopeptide transporters and their mechanisms of action.

## MATERIALS AND METHODS

### Bacterial strains and growth conditions.

The pneumococcal strains utilized in this study are described in Table 2. Strains were cultured on sheep blood agar (BA) or in Todd-Hewitt medium with 0.5% yeast extract (THY) and incubated at 37°C with 5% CO_2_. Strains were grown to mid-log phase and collected for storage at −80°C in 1-mL aliquots containing 20% glycerol or were directly utilized in the assay.

**TABLE 2:** Pneumococcal strains used in this study

Strain	Description	Antibiotic resistance marker (concn [μg/ML])^*a*^	Reference
MNZ41	NESp carriage isolate	Tmp (50)	11
JLB01	MNZ41 Δ*aliC* Δ*aliD*	Spec (300)	11
JLB02	MNZ41 Δ*aliC*	Kan (500)	11
JLB04	MNZ41 Δ*aliD*	Spec (300)	11
JLB10	MNZ41 Δ*cbpAC*	Spec (300)	11
SPJV40	Serotype 38 pneumonia isolate	ND	44
CDT11	SPJV40 Δ*aliD*	Erm (1)	This study
CDT14	JLB10/pABG5::*cbpAC*	Kan (250)	This study

a Tmp, trimethoprim; Spec, spectinomycin; Kan, kanamycin; ND, not determined; Erm, erythromycin.

### DNA mutagenesis.

Specific details of pneumococcal strain origins and mutagenesis have been described previously (11, 44). In brief, the MNZ41 AliC/AliD double mutant (JLB01) was created by the allelic replacement of *cps* with a spectinomycin resistance cassette. The MNZ41 AliC (JLB02) and AliD (JLB04) single mutants were generated by the allelic replacement of *aliC* or *aliD* with a kanamycin resistance cassette and a spectinomycin resistance cassette, respectively. The MNZ41 CbpAC mutant (JLB10) was generated by the allelic replacement of *cbpAC* with a spectinomycin cassette. Mutants were confirmed by sequencing. A complemented strain of the Δ*cbpAC* mutant was generated by cloning the *cbpAC* gene amplified from MNZ41 genomic DNA with primers F_cpbAC (GCCGGAATTCATGAATAAGAAAAAAATGATTTTAACAAGTC) and R_cbpAC (GCCGCCTGCAGGTTAAACCCATTCACCATTGG) into plasmid pABG5. The amplified DNA and plasmid were digested with EcoRI and SbfI or EcoRI and PstI, respectively, and ligated before transformation into Escherichia coli DH5α competent cells. Plasmids were verified by sequencing and transformed into deletion strain JLB10. The deletion of the *aliD* gene from SPJV40 was performed by in-frame allelic replacement with an erythromycin resistance cassette. Flanking regions were amplified with primers KLO_13 (AAGTGGCGTCTCGTCAAATTCCTGACGAGAAGGTAG) and KLO_14 (AAGTGGCGTCTCGCTAATAGTGGGAATTTGTAAAGTTAATTG) for the upstream fragment and KLO_17 (AAGTGGCGTCTCGAACTTTTTCAAGGAGAATTATAAAGACA) and KLO_18 (AAGTGGCGTCTCGTTGATATTGCCCATCAGCTG) for the downstream fragment. The erythromycin cassette was amplified from ΔPAC using primer pair KLO_15 (AAGTGGCGTCTCCTTAGTAGGAGGAAAATTAATGAACAAAA) and KLO_16 (AAGTGGCGTCTCCAGTTATTTCCTCCCGTTAAATAATAGAT). A BsmBI restriction enzyme site was added to the primer (indicated by underlining in the sequences) for GoldenGate assembly of the three fragments. The transformation of pneumococcal strains was performed in competence medium (THY medium, 0.2% bovine serum albumin [BSA], 0.2% glucose, 0.2% CaCl_2_). Strains were grown to an optical density at 600 nm (OD_600_) of 0.1, and competence was induced with 100 ng of competence-stimulating peptide 1 (CSP1) and 100 ng of CSP2 before the addition of the DNA construct (100 ng). Transformants were selected on BA supplemented with the appropriate antibiotics, and mutants were verified by sequencing (Table 2).

### RNA extraction and RT-qPCR analysis.

The bacterial cultures utilized in the reverse transcription-quantitative PCR (RT-qPCR) studies were suspended in a 2-fold volume of RNAprotect bacterial reagent (Qiagen), centrifuged, and stored at −20°C. Total RNA was extracted using the RNeasy Plus minikit (Qiagen) according to the manufacturer’s protocol. DNA was removed using 1 U of DNase I (Promega) at 37°C for 30 min, followed by incubation with DNase stop solution (Promega) at 65°C for 10 min. The RNA integrity was verified by gel electrophoresis, and the concentration was obtained using a NanoDrop spectrophotometer (Thermo Scientific). cDNA was generated with iScript reverse transcription supermix (Bio-Rad) and used as a template for RT-qPCRs. RT-qPCR was carried out using the PerfeCTa SYBR green SuperMix kit (Quanta Bio) and a CFX96 real-time PCR detection system (Bio-Rad). RT-qPCR analysis of the *cbpAC* expression levels of MNZ41 and its mutant derivatives was performed using primers F_RT_cbpAC (CATCAAAAAAGCTGCCGAAG) and R_RT_cbpAC (CAACAATTCCACCTAATTGCG). Analyses of the *pspA* expression levels of SPJV40 and its *aliD* mutant were performed using primers F_RT_pspA (CACCAAAACCAGAGAAGCC) and R_RT_pspA (ATCCTGTCGCCATTGAACC). Melting curves were generated by a cycle of 95°C for 3 min followed by 40 cycles of 95°C for 15 s, 58°C for 30 s, and 72°C for 30 s. The relative mRNA expression level was normalized to the constitutive expression level of the *gyrA* gene (primers F_RT_gyrA [CCCATAGTTGCACGTCCTGT] and R_RT_gyrA [TCGTGGTGGTAAGGGAATGC]) and calculated by the comparative threshold cycle (*C_T_*) (2^−ΔΔ^*^CT^*) method for two independent biological replicates performed in triplicate (45).

### Flow cytometry.

Pneumococcal cultures grown to mid-log phase at an OD of 0.2 were pelleted, washed twice with phosphate-buffered saline (PBS), and suspended in gelatin-Veronal buffer (GVB) (0.1% gelatin, 5 mM Veronal, 145 mM NaCl [pH 7.3]) containing 20% pooled normal human serum (NHS; Fisher BioReagents). Samples were incubated with rotation for 1 h at 37°C and subsequently washed twice with PBS. Pneumococci were collected and incubated with rotation with fluorescein isothiocyanate (FITC)-conjugated mouse anti-human C3 (Cedarlane Laboratories Limited) (1:500) for 1 h at 37°C in the dark. Cells were collected, washed with PBS, and suspended in 1 mL PBS. Cells were analyzed at 50,000 gated events by a NovoCyte flow cytometer to determine C3 deposition. Serotype 38 strain SPJV40 and WT NESp strain MNZ41 cells that were not incubated with NHS were used as negative controls.

To determine CRP deposition, a protocol similar to the one described above was performed. Pelleted cells were suspended in GVB containing 50% NHS. After incubation and subsequent washes, samples were incubated with FITC-conjugated goat anti-human CRP (Bethyl Laboratories, Inc.) (1:500). Serotype 38 strain SPJV40 and WT NESp strain MNZ41 cells that were not incubated with NHS were used as negative controls. Figure S1 in the supplemental material demonstrates the gating strategy used for the analysis. Analysis of the flow cytometry results was done with a minimum of three independent replicates.

10.1128/mbio.03325-22.2 FIG S1 Gating strategy for analysis. Forward and side scatterplots were initially used to gate the dense bacterial population (P1). Further gating of Forward Scatter-Area and Forward Scatter-Height was used to correct and remove values from large chains of bacteria (P2). A histogram containing data for bacteria within both the P1 and P2 gates was used to determine the percentage of positive cells after antibody incubation. (A) Sample of bacteria not incubated with serum with a gate containing all nonstained cells. (B) Example of a sample incubated with serum with the same gating strategy and percent positive cells binding the serum protein of interest. Download FIG S1, TIF file, 2.9 MB. Copyright © 2023 Thompson et al. 2023 Thompson et al. https://creativecommons.org/licenses/by/4.0/ This content is distributed under the terms of the Creative Commons Attribution 4.0 International license.

### LC-MS/MS analysis.

Complement component binding was determined by spectral counts using tandem mass spectrometry (MS/MS). Proteome profiling by spectral counting was used to determine human plasma proteins that bound WT MNZ41 compared to JLB01 (Δ*aliC* Δ*aliD*). Strains were incubated in pooled human plasma. Samples were extensively washed, and bound proteins were removed and prepared for spectral analysis. Protein samples were run on a short SDS-PAGE gel as described in a previously published protocol (46). Proteins in gel bands were reduced with dithiothreitol (DTT) (Sigma) and alkylated with iodoacetamide (IAA) (Sigma). The gel bands were then washed, dried, and rehydrated with a buffer containing porcine trypsin (Promega). Samples were digested overnight and acidified, and the peptides were extracted. The extracts were dried and reconstituted in 5% formic acid. The peptide samples were loaded onto a nanoscale capillary reverse-phase C_18_ column using a high-performance liquid chromatography (HPLC) system (Easy-nLC 1000; Thermo) and eluted by a gradient. The eluted peptides were ionized and detected using an in-line mass spectrometer (LTQ Orbitrap Elite; Thermo). The MS and MS/MS spectra were collected over a 90-min liquid chromatography gradient. MS spectra were collected first, and the 20 most abundant ions were sequentially isolated for MS/MS analysis. The process was cycled over the entire liquid chromatography gradient. Database searches were performed using the Sequest (version 28, revision 13) (47) search engine against a composite target/decoy UniProt human protein database (48). All matched MS/MS spectra were filtered by mass accuracy and matching scores to reduce the protein false discovery rate to <1%. Spectral counts matching to individual proteins reflect their relative abundance in one sample after the protein size is normalized. The spectral counts between samples for a given protein were used to calculate the *P* value, which was derived by a *G* test (49). *P* values of <0.05 indicate significant changes.

### *In vivo* infection model.

C57BL/6J and C1qa^−/−^ [B6(Cg)-C1qatm1d(EUCOMM)Wtsi/TennJ] mice were purchased from the Jackson Laboratory. Male and female 10- to 12-week-old wild-type and C1q^−/−^ mice were utilized in murine studies. Mice were challenged intraperitoneally (i.p.) with 200 μL of PBS containing 5 × 10^8^ CFU of MNZ41 and the isogenic mutant strains JLB01 (Δ*aliC* Δ*aliD*) and JLB10 (Δ*cbpAC*) to determine bacterial systemic survival and murine mortality following bacteremia. Murine survival was monitored over a 5-day period. Bacterial systemic survival was evaluated using pneumococci collected through retro-orbital bleeding at 4, 24, 48, 72, 96, and 120 h postinfection. The gathered blood samples (100 μL) were serially diluted and plated onto BA to enumerate the CFU. A minimum of four mice per strain were used for each time point in two independent experiments (*n* = 8), except where mortality precluded bacterial quantification.

### Cell culture.

The human promyelocytic leukemia cell line HL-60 (ATCC CCL-240) was obtained from the American Type Culture Collection (ATCC). HL-60 cells were maintained in Iscove’s modified Dulbecco’s medium (IMDM; Gibco) supplemented with 20% heat-inactivated fetal bovine serum (FBS), 100 U/mL penicillin, and 1 mg/mL streptomycin and cultured in an incubator at 37°C with 5% CO_2_. Cells were routinely counted to maintain a density of between 10^5^ and 10^6^ cells/mL. HL-60 cells at a density of 10^6^ cells/mL were differentiated into neutrophil-like cells using growth medium supplemented with 1.25% dimethyl sulfoxide (DMSO) and incubated for a period of 5 to 7 days. Prior to use in the assay, the cells were washed in IMDM, and viable cells were counted based on trypan blue exclusion using a hemocytometer. HL-60 cells were then suspended in IMDM plus 1% BSA at a concentration of 2 × 10^5^ cells/mL for use in the opsonophagocytic killing assay (OPA).

### Opsonophagocytic killing assay.

The pneumococcal strains utilized in the opsonophagocytic killing assay were grown to mid-log phase at an OD of 0.2 and diluted to a concentration of 5 × 10^3^ CFU/mL in Hanks’ balanced salt solution (HBSS) plus 0.1% gelatin. One-hundred-microliter reaction mixtures were preopsonized with 3% normal mouse serum (NMS) or C1q-deficient serum and incubated with rotation for 30 min at 37°C. After incubation, 100 μL of differentiated HL-60 cells (2 × 10^5^ cells/mL) was added to the preopsonized cultures, or IMDM with 1% BSA was added to the controls. Samples were incubated with rotation at 37°C for 45 min. The percentage of killing was determined by dribble plating the samples in duplicate onto sheep BA plates and calculated in comparison to the control under the same conditions.

### Ethics statement.

Animal studies were performed in accordance with submitted protocols approved by the Institutional Animal Care and Use Committee (IACUC) of the University of Mississippi Medical Center. Animal care and experimentation adhered to the Animal Welfare Act (Public Law 89-544) and its amendments and Public Health Service guidelines for the humane care and use of laboratory animals (50).

### Statistical analysis.

Survival data were analyzed by the Mantel-Cox log rank test using Prism 5 software (GraphPad Software, Inc., San Diego, CA). Flow cytometry statistics were performed using NovoExpress software (ACEA Biosciences, San Diego, CA). Percentages of binding to serum proteins were analyzed by a *t* test using Prism 5 software. Opsonophagocytosis survival experiments were analyzed by one-way analysis of variance (ANOVA) followed by a Bonferroni *post hoc* test using Prism 5 software. A *P* value of <0.05 was considered to be statistically significant.

10.1128/mbio.03325-22.1 TEXT S1 Microscopic analysis of capsule. Download Text S1, DOCX file, 0.02 MB. Copyright © 2023 Thompson et al. 2023 Thompson et al. https://creativecommons.org/licenses/by/4.0/ This content is distributed under the terms of the Creative Commons Attribution 4.0 International license.
