# Supplementary material for: Oligopeptide Transporters of Nonencapsulated Streptococcus pneumoniae Regulate CbpAC and PspA Expression and Reduce Complement-Mediated Clearance
Source: mBio. 2023 Jan 10;14(1):e03325-22. doi: 10.1128/mbio.03325-22 (PMC9973307; doi:10.1128/mbio.03325-22)
Supplement: TEXT S1 [file mbio.03325-22-s0001.docx]

**SUPPLEMENTARY MATERIAL**

**MATERIAL/METHODS**

**Microscopic Analysis of Capsule.** Negative staining of *S. pneumoniae* serotype 38 WT and AliD mutant strains was conducted using nigrosin stain. Nigrosin stain was prepared by dissolving 10% water soluble nigrosin (Alfa Aesar) in distilled water in a boiling water bath for 30 minutes. The water lost by evaporation was replaced, and 0.5% formalin was added to the solution. After filtering through filter paper twice, a single drop of nigrosin stain was added onto a clean microscope slide. Using sterile technique, overnight broth cultures of serotype 38 WT (SPJV40) or AliD mutant (CDT11) strains were mixed 1:1 with the nigrosin stain on the slide and smeared into a film using another clean microscope slide. The smear was air dried and subsequently saturated with crystal violet for 1 minute. The slide was gently rinsed with water and allowed to dry before visualization. The samples were examined by light microscopy, and the presence of capsule was assessed. Capsule thickness was determined by measuring cross-sectional areas of at least 15 randomly chosen bacteria from midcell to the edge of the capsule. Captured images were analyzed with ImageJ software.
